# Combating ozone stress through N fertilization: A case study of Indian bean (*Dolichos lablab* L.)

**DOI:** 10.3389/fpls.2023.1125529

**Published:** 2023-02-22

**Authors:** Ansuman Sahoo, Parvati Madheshiya, Ashish Kumar Mishra, Supriya Tiwari

**Affiliations:** Laboratory of Ecotoxicology, Centre of Advanced Study in Botany, Institute of Science, Banaras Hindu University, Varanasi, India

**Keywords:** nitrogen amendments, ozone, antioxidants, stomatal conductance, dolichos

## Abstract

The present study investigates the efficiency of nitrogen (N) amendments in the management of ozone (O_3_) stress in two varieties (Kashi Sheetal and Kashi Harittima) of Indian bean (*Dolichos lablab* L.). Two O_3_ concentrations, ambient (44.9 ppb) and elevated (74.64 ppb) were used, and each O_3_ concentration has 3 nitrogen (N) dose treatments viz recommended (N1), 1.5 times recommended (N2), 2 times recommended (N3) and no nitrogen, which served as control (C). The experiment concluded Kashi Sheetal as O_3_ tolerant, as compared to Kashi Harittima. N amendments were effective in the partial amelioration of O_3_ stress, with N2 being the most effective nitrogen dose, at both ambient and elevated O_3_ concentrations. Kashi Sheetal has been determined to be O_3_ tolerant due to greater endogenous levels of H_2_O_2_ accumulation and enzymatic antioxidant contents with O_3_ exposure. The O_3_-sensitive variety, Kashi Harittima, responded more positively to N treatments, at both O_3_ concentrations. The positive effect of N amendments is attributed to the stimulated antioxidative enzyme activity, rather than the biophysical processes like stomatal conductance. Strengthened defense upon N amendments was attributed to the enhanced activities of APX and GR in Kashi Sheetal, while in Kashi Harittima, the two enzymes (APX and GR) were coupled by SOD and CAT as well, during the reproductive phase. Yield (weight of seeds plant^-1^) increments upon N (N2) amendments were higher in Kashi Harittima (O_3_ sensitive), as compared to Kashi Sheetal (O_3_ tolerant) at both ambient and elevated O_3_ concentration, due to higher antioxidant enzymatic response and greater rate of photosynthesis in the former.

## 1 Introduction

Over the past few decades, speedy industrial growth and unrestrained urbanization in developing nations have greatly increased the concentration of primary and secondary pollutants in the atmosphere (Dhevagi et al., 2021). The concentration of phytotoxic secondary pollutant tropospheric ozone (O_3_) depends on the levels of certain primary air pollutants such as nitrogen oxides (NO_x_), and volatile organic compounds (VOCs). In recent years, tropospheric ozone has shown a severe impact on plants because of its high oxidative potential (Duque et al., 2021). Background O_3_ concentration in the troposphere has increased by 36% since pre-industrial times (Mukherjee and Hazra, 2022). Nearly one-quarter of the world is currently at risk of high O_3_ and it is predicted that the levels could rise to 20% by 2050 (Meehl et al., 2018). Because of chemical interactions between O_3_ precursors, industrial pollutants, and sunlight, surface ozone levels are increasing at a rate of 0.5 to 2.5 percent each year (Wang et al., 2022). Because it has a negative impact on people’s health, plants, and the ecosystem worldwide, the rising concentration of ground-level O_3_ has become a global issue (Mills et al., 2013). Even if strict adherence to the air quality regulations of 2000 is maintained, the O_3_-induced damage is projected to worsen, according to future emission scenario legislation taking emissions for the year 2030 (CLE-2030) (Van Dingenen et al., 2009). A number of modeling studies have depicted high tropospheric ozone concentration in the future thus making the study of the impact of elevated ozone on plants even more important.

The stomata are the primary entry point for ozone into plant leaves (Stella et al., 2013). Ozone quickly reacts with intracellular components and forms reactive oxygen species (ROS) such as hydrogen peroxide (H_2_O_2_), superoxide radicals (O^2-^), and hydroxyl radicals (OH**^·^**) inside the leaf (Zhang et al., 2017). These ROS enhance the antioxidant defense response downstream, which mitigates the fatal consequences of accumulating ROS. Contradictorily, when ROS generation surpasses the antioxidant efficiency, plants show ozone vulnerability in the form of foliar injury symptoms, alteration in metabolic processes, yield loss, and other ozone-related effects (Singh et al., 2014; Singh et al., 2018; Hayes et al., 2020). Excessive ROS levels within the cells brought on by O_3_ damage the membranes (lipid peroxidation), oxidize proteins, degrade RNA and DNA, enhance the production of secondary metabolites, degrade chlorophyll, and ultimately result in apoptosis (Choudhury et al., 2017). However, a vast variety of antioxidants and ROS-scavenging enzymes such as catalase (CAT), superoxide dismutase (SOD), glutathione reductase (GR), and ascorbate peroxidase (APX), produced by cells upon O_3_ exposure to attenuate the impacts of ROS in the cells (Picchi et al., 2017). In order to maintain steady development in plants and to aid in the damage repair caused by various stressors, nutrient supplements have frequently been utilized (Sahoo and Tiwari, 2021). Nitrogen (N) is the fourth most common element in living things and is theoretically an important component that supports plant development (Sahoo and Tiwari, 2021). Nitrogen amendment not only maintains greater protein levels it can also be used for repair processes and facilitates the remobilization of nutrients to reproductive parts, sustaining better production (Zeng et al., 2017). Nitrogen applications tend to improve the photosynthetic potential of the plant which can be commonly coupled with stomatal conductance (Zhang et al., 2018). The addition of nitrogen may partly counteract the detrimental effects of O_3_ on a few plants’ morphological characteristics (Yendrek et al., 2013). Plants can either strengthen their antioxidant defense against an O_3_-induced ROS surge, fix more carbon to use for O_3_-induced injury, or direct more biomass into reproductive organs to sustain greater yields in response to N amendments (Podda et al., 2019).

A significant portion of India’s protein needs is fulfilled by pulses, one of the country’s essential crops, which are grown on an area of 28.3 million hectares and produced approximately 25.7 million metric tonnes in the fiscal year 2020–21 (Statista, 2021). In Asia, where the mean ambient ozone concentration ranged between 35 and 75 ppb during the growing season of the crop, legumes had a substantial production loss of 10 to 66 percent (Emberson et al., 2009). *Dolichos lablab* L. is a versatile crop, several plant components may be consumed as food, animal feed, and green manure (Davari et al., 2018; Rana et al., 2021). It is a low-cost source of protein and micronutrients when compared to other legumes, highlighting the importance of its study in view of food security and nutrition in near future (Letting et al., 2021). These micronutrients, which play a significant role in the diets of resource-limited households in rural areas, include phosphorus, fiber, niacin, and thiamine (Letting et al., 2021). The most recent study shows how efficient lablab bean extracts are at preventing the spread of viral illnesses like influenza and SARS-CoV-2, which has been called a global pandemic (Liu et al., 2020). To the best of our knowledge, the sensitivity of *Dolichos lablab* L. varieties towards tropospheric ozone has not been defined. The present work is the first effort to characterize the sensitivity of two varieties of *Dolichos lablab* L. towards O_3_ stress. In addition, it also exemplifies the efficiency of nitrogen amendments in the management of O_3_ stress and the mechanistic approach adopted therein. Through our work, we hypothesize that the ameliorative effect of N amendments in O_3_-stressed plants is mostly credited to the antioxidant response rather than the biophysical parameters like stomatal conductance.

## 2 Materials and methods

### 2.1 Experimental area

The area of study was the Botanical Garden at Banaras Hindu University, Varanasi. It is a sub-urban site located in the Indian subcontinent at 25^0^14’ N latitude, 82^0^03’ E longitude, and 76.19 m above sea level. The crop was grown during the winter season from November 2021 to March 2022. A subtropical humid climate prevails in the area, with distinctive summer, rainy, and winter seasons. The soil is sandy loam in texture (sand 45%, silt 28%, clay 27%) having an organic carbon content of 0.67%, pH 7.4, nitrogen content of 0.12%, and phosphorus content of 0.065%.

### 2.2 Plant material

Two varieties of Indian bean (*Dolichos lablab* L.) namely, Kashi Sheetal and Kashi Harittima were used for the experiment. The seeds of the above varieties were procured from the Indian Institute of Vegetable Research, Varanasi (IIVR-IARI). Kashi Sheetal is a semi-pole type variant with low-temperature tolerance. It is very rich in protein and can give a yield of about 18-20 tonnes/hectare. The color of flowers of this variety is violet and the pods were green with a dark red lining at their edges. Kashi Harittima is reasonably resistant to Dolicho’s yellow mosaic virus and also exhibits tolerance against aphids and pod borers. It is a high-yielding variety with parchment-free green-colored pods and white flowers.

### 2.3 Experimental design

**Figure 1:**
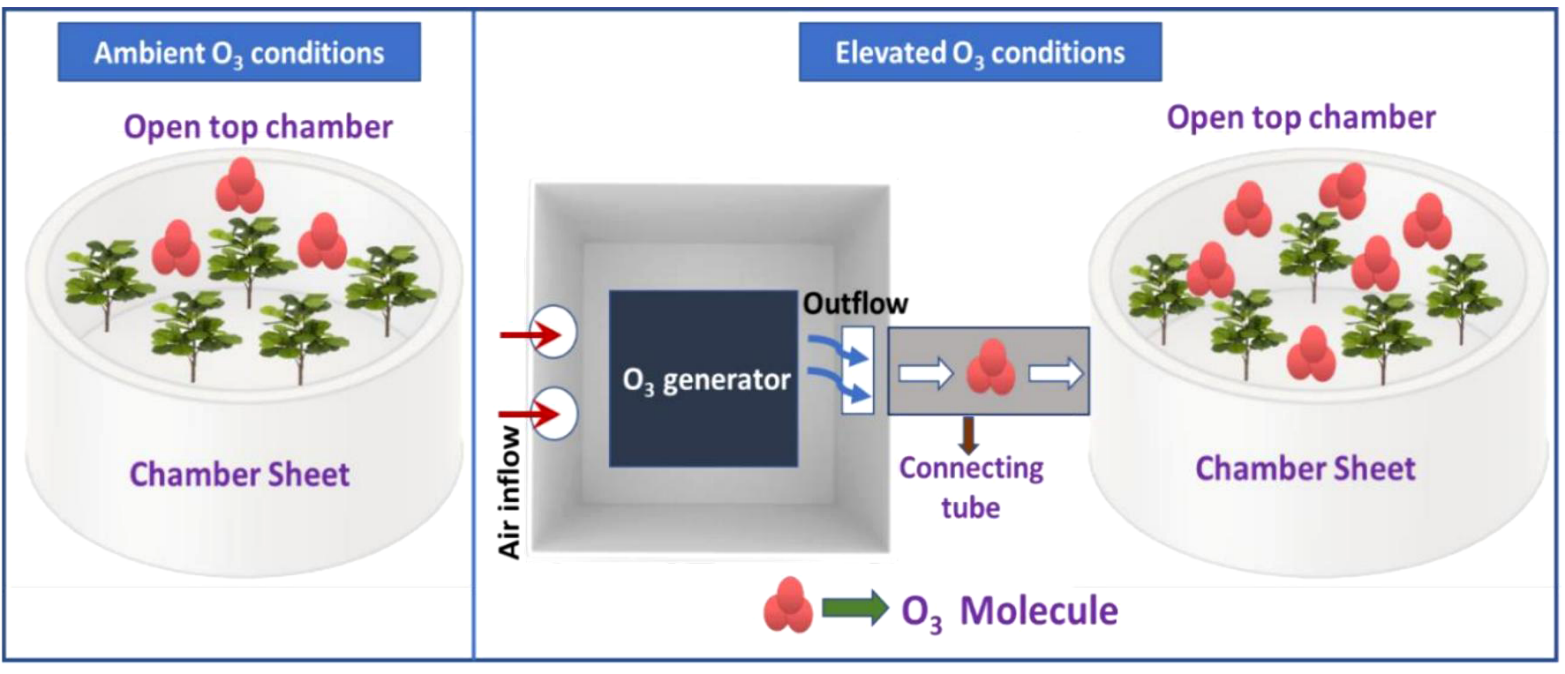
Diagrammatic representation of experimental setup.

**Figure 2:**
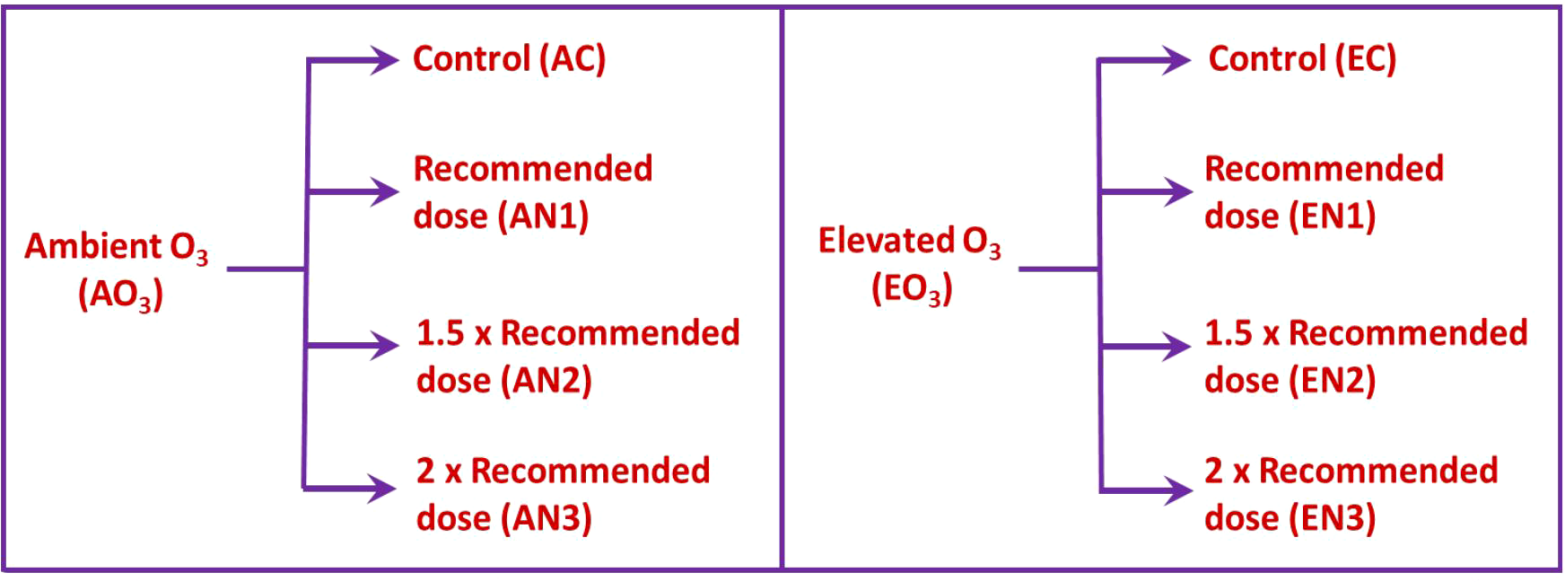
The different patterns of nitrogen amendments under ambient and elevated O_3_ conditions.

The study was carried out by installing custom-built open-top chambers (OTCs) in the experimental area under ambient field circumstances. The diameter of the OTCs was 1.5m and the height was 1.8m. OTCs were set up in the botanical garden and were categorized as ambient O_3_ OTCs and elevated O_3_ OTCs (ambient + 30 ppb) on the basis of O_3_ concentrations maintained in them. The experimental setup is explained in **Figure 1**. Each level of O_3_ treatment was further supplemented with 3 doses of nitrogen amendments viz recommended (AN1 and EN1), 1.5 times recommended (AN2 and EN2) and 2 times recommended (AN3 and EN3). For each O_3_ treatment, control was also maintained (AC and EC), wherein no N treatment was given (**Figure 2**). Each treatment was replicated thrice. The nitrogen fertilizer was applied to the soil and the treatment was given in two phases i.e, one in the vegetative phase and the other in the reproductive phase. In OTCs, seeds were hand sown in accordance with standard agricultural procedures. Ozone was produced by the ozone generator (A1G, Faraday, India) and then transported through a connecting tube to the OTCs designated for elevated O_3_. For ozone formation, the generator utilized high-frequency corona discharge technology. The release of ozone into the growth chamber was set at multiple points at the base of the OTCs. The experimental setup and design are displayed in **Figure 1**.

### 2.4 Ozone monitoring

Continuous O_3_ monitoring was done throughout the experiment. The concentrations of elevated ozone were standardized once the OTCs were mounted in the field. Ozone generators were calibrated to create the necessary levels of elevated ozone (ambient + 30 ppb). Using an automated real-time O_3_ monitoring instrument (Model APOA 370, HORIBA Ltd., Japan), eight-hour O_3_ monitoring (9:00-17:00 h) was carried out during the growth period.

### 2.5 Histochemical localization of H_2_O_2_

By using a histochemical analysis, the test plants’ flag leaves in the reproductive phase were studied *in-situ* localization of accumulated hydrogen peroxide (H_2_O_2_). For each cultivar, three randomly chosen leaf samples from different treatments were taken. The procedure given by Thordal-Christensen et al. (1997) was followed for studying the *in-situ* localization of H_2_O_2_ in leaves using 3, 3′-Diaminobenzidine (DAB).

### 2.6 Physiological parameters

Photosynthetic rate (A), stomatal conductance (g_s_), and internal CO_2_ concentration (Ci) were analyzed by using a portable photosynthetic instrument (CIRAS-3, PP SYSTEMS). All these parameters were measured for both ambient and elevated conditions. Three randomly chosen plants per plot had their fourth fully grown leaf from the top examined for physiological parameters. At 40 DAG and 60 DAG, measurements were made between 9:00 and 10:30 hours on cloud-free days. The instrument was calibrated utilizing a known source of CO_2_ set at 510 ppm and the photosynthetic active radiation (PAR) set at 1200 mmol m^-2^ s^-1^.

### 2.7 Enzymatic antioxidants and lipid peroxidation

For the enzymatic assay, 0.2 gm of fresh weight of leaf was taken and homogenized using liquid nitrogen. 5 ml of extracting buffer was used to homogenize the leaf tissues in order to extract antioxidant enzymes. The buffer was prepared using 1 M phosphate buffer of pH 7.0 of polyvinylpyrrolidone (PVP), phenyl methane sulfonyl fluoride (PMSF), Ethylenediaminetetraacetic acid (EDTA), and Triton-X-100 at 4°C. Antioxidant enzymes like superoxide dismutase (SOD), ascorbate peroxidase (APX), catalase (CAT), and glutathione reductase (GR) were estimated by the protocols (Fridovich, 1975; Nakano and Asada, 1981; Aebi, 1984; Anderson, 1996). The method developed by Heath and Packer (Heath and Packer, 1968) was used to estimate the malondialdehyde (MDA) content which represents lipid peroxidation. Leaf tissues (0.5 g) were extracted in 5% TCA, and 4 mL of 20% TCA containing 0.5% TBA was added. Following centrifugation, the sample mixture’s absorbance was measured at 532 and 600 nm.

### 2.8 Yield

In order to determine yield, five plants per OTC were harvested in the first week of March and quantified for yield attributes. The number of seeds plant^-1^, the weight of seeds plant^-1^, and the test weight per 1000 seeds were estimated for each treatment.

### 2.9 Statistical analysis

All the statistical tests were executed using SPSS software (SPSS Inc. version 25.0, IBM Corp, New York). The three-way ANOVA was performed to examine the significance of the age of sampling, nitrogen treatment, and varieties of beans. Using the Shapiro-Wilk test, the normality of each dataset was examined, and the distribution was determined to be normal in all instances since the P values were all over the threshold of significance (0.05). For both cultivars, the data was examined *via* principal component analysis (PCA). The correlation matrix and regression technique were used to perform the PCA.

## 3 Results

### 3.1 O_3_ monitoring

**Figure 3:**
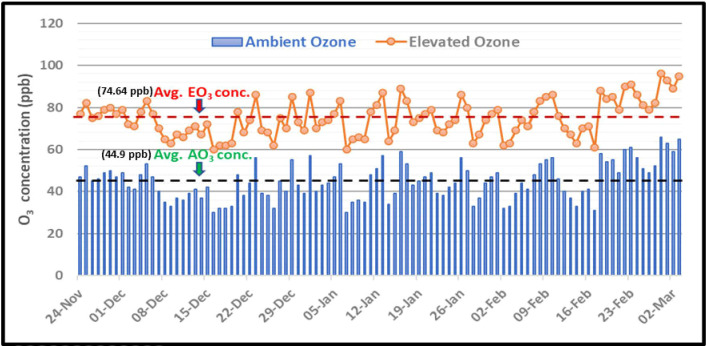
Mean 8h O_3_ concentrations during the growth period of Hyacinth beans.

During the growth period of the crop, the mean concentration of ambient O_3_ and elevated O_3_ was found to be 44.9 ppb and 74.64 ppb, respectively. The ambient O_3_ concentration ranged from 29 ppb to 66 ppb and the elevated O_3_ concentration ranged between 58 ppb to 97 ppb (**Figure 3**).

### 3.2 Histochemical localization

**Figure 4:**
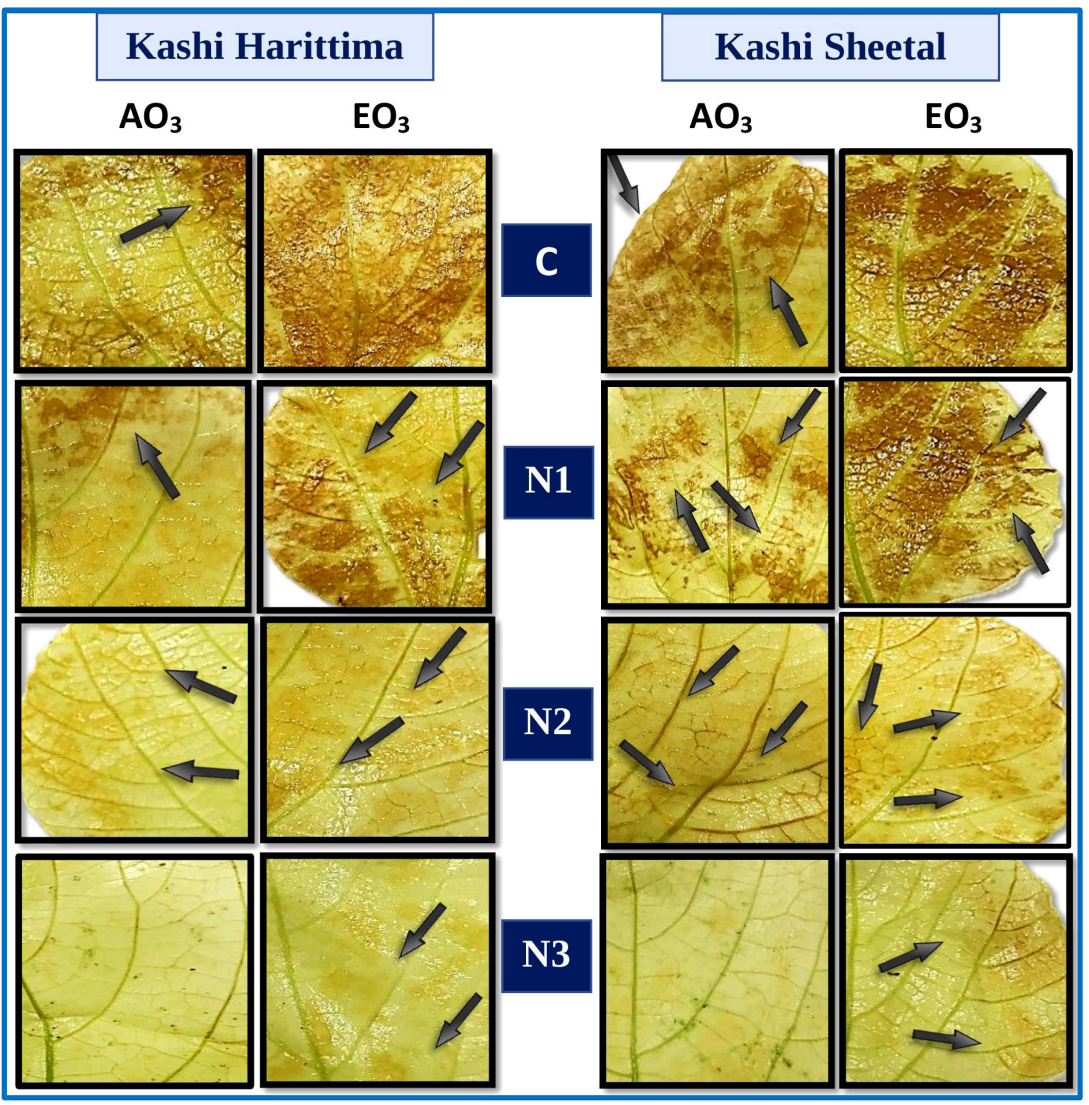
Histochemical localization of hydrogen peroxide (H_2_O_2_) stained with DAB (brown color) in two varieties of *Dolichos lablab* L. exposed to AO_3_ and EO_3_ under different nitrogen fertilization levels.

Histochemical detection of H_2_O_2_ in the leaves of both varieties of *Dolichos lablab* L. indicated the presence of H_2_O_2_ (**Figure 4**). The interaction of H_2_O_2_ with DAB under elevated O_3_ (EO_3_) as compared to ambient O_3_ (AO_3_) produced a stronger reddish-brown stain in the leaf tissues of plants of both varieties. As compared with Kashi Harittima, leaves of Kashi Sheetal contained higher concentrations of H_2_O_2_. Furthermore, as nitrogen amendment amounts are increased, the H_2_O_2_ content of both varieties of Indian bean decreased significantly.

### 3.3 Lipid peroxidation and antioxidative enzymatic activity

**Figure 5:**
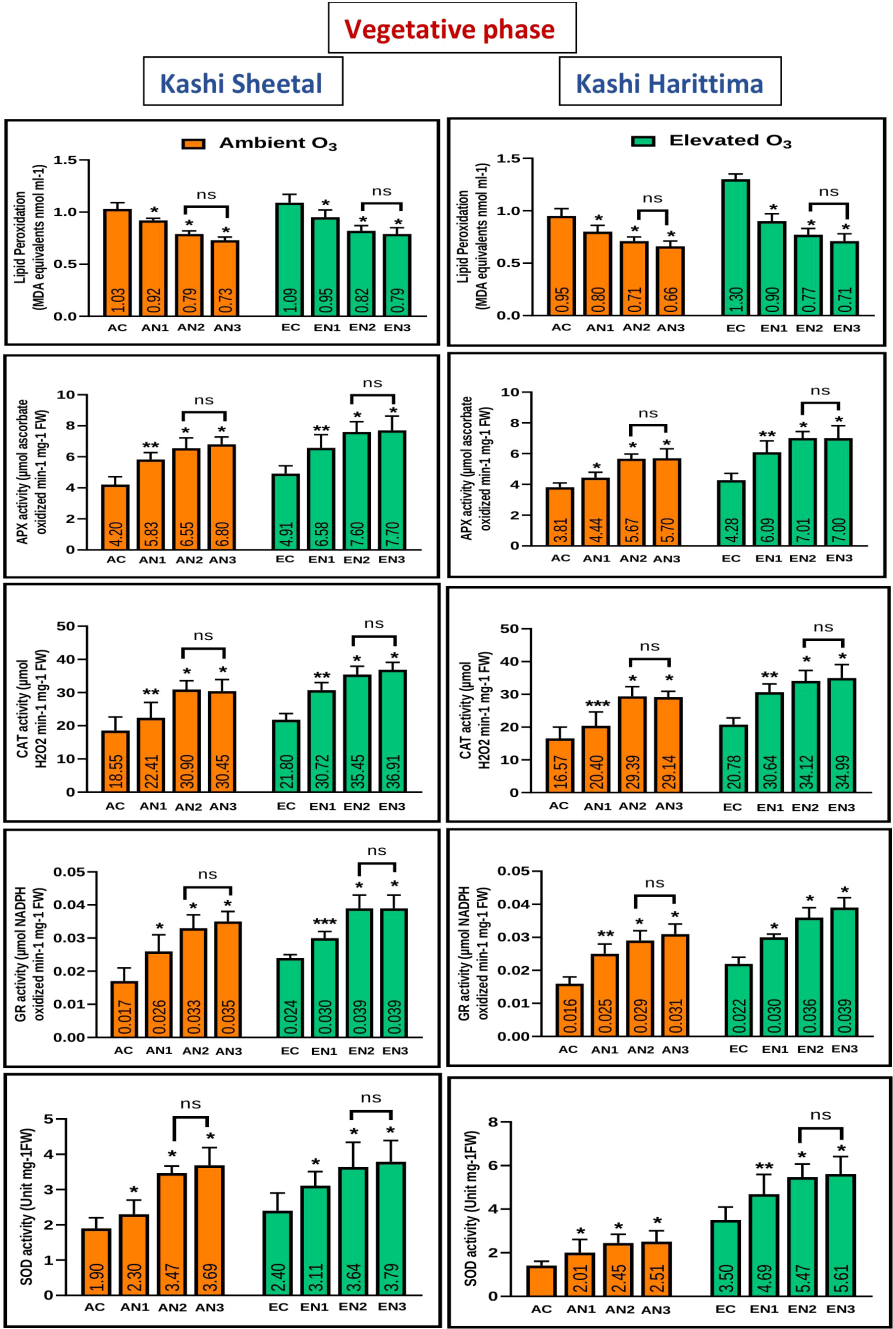
Variations in antioxidative enzymes in both varieties of *Dolichos lablab* L grown under AO_3_ and EO_3_ in the vegetative phase. Values are mean ± SE. Level of significance between AO_3_ and EO_3_ treated plants based on t-test; ns; not significant; *, P ≤ 0.05; **, P ≤ 0.01; ***, P ≤ 0.001.

**Figure 6:**
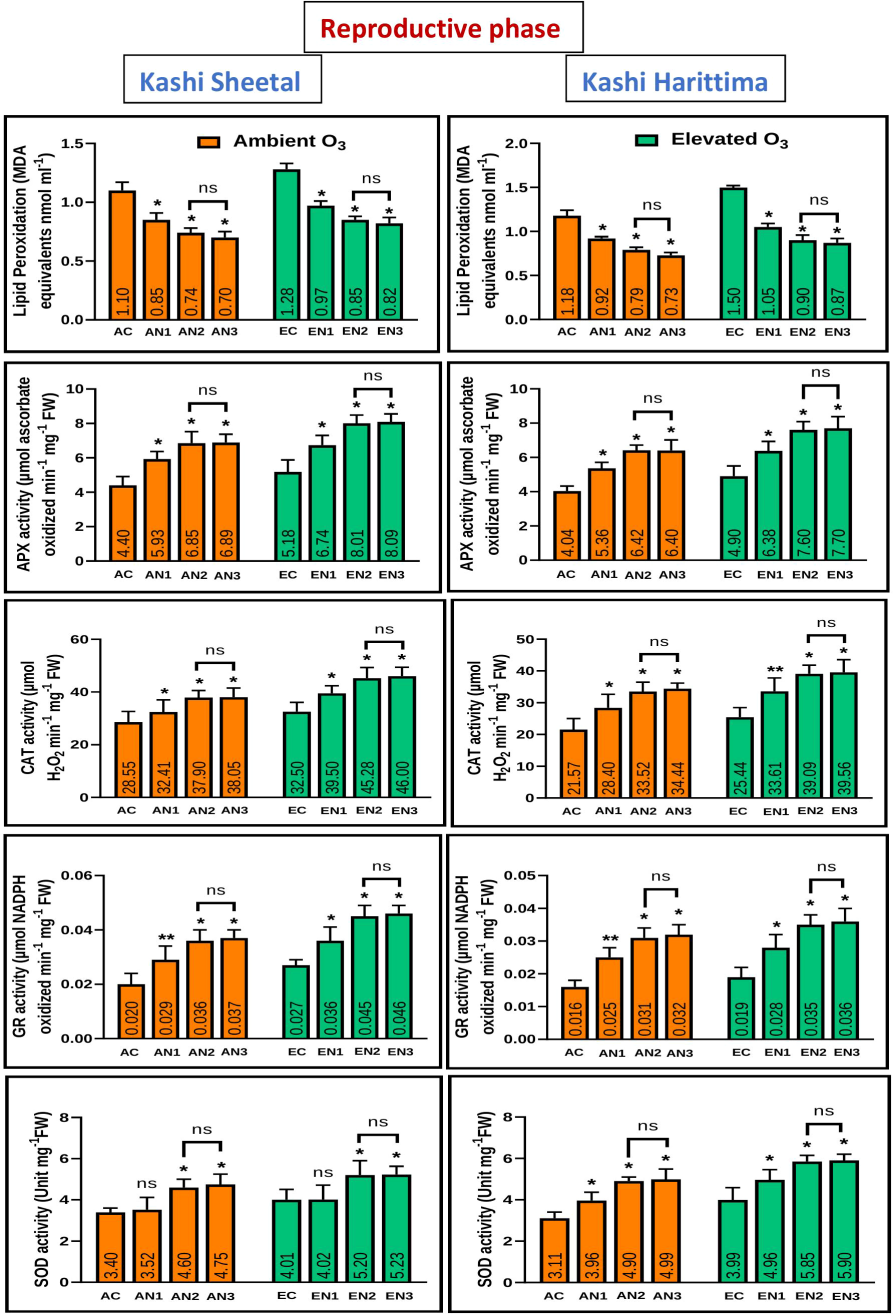
Variations in antioxidative enzymes in both varieties of *Dolichos lablab* L grown under AO_3_ and EO_3_ in the reproductive phase. Values are mean ± SE. Level of significance between AO_3_ and EO_3_ treated plants based on t-test; ns; not significant; *, P ≤ 0.05; **, P ≤ 0.01; ***, P ≤ 0.001.

Plants grown in elevated O_3_ conditions showed significantly higher levels of lipid peroxidation (LPO) in their leaves than plants grown in ambient O_3_ conditions in both varieties of *Dolichos lablab* L. (**Figures 5**, **6**). All three types of nitrogen treatments (N1, N2, and N3) showed significantly decreased lipid peroxidation as compared to control in both Kashi Sheetal and Kashi Harittima. For both varieties, the degree of lipid peroxidation was higher during the reproductive period than during the vegetative phase (**Figures 5**, **6**). In both the vegetative and reproductive stages, Kashi Harittima plants had increased lipid peroxidation than Kashi Sheetal plants. The differences in the level of lipid peroxidation between treatments N2 and N3 were non-significant for both phases of growth. The results of the four-way ANOVA show that LPO varied significantly due to two individual factors such as age and treatment. Significant variations in LPO were also observed due to interactions of age with variety, ozone, and treatment factors (**Table 1**).

**Table 1:** F-ratio and level of significance of selected biochemical and physiological characteristics of *Dolichos lablab* L.

	LPO	APX	GR	CAT	SOD	A	g_s_	Ci
AGE	**	***	**	ns	ns	***	***	***
OZONE	ns	***	***	***	***	***	***	***
VARIETY	ns	***	***	***	***	***	***	***
TREATMENT	***	***	***	***	***	***	***	***
AGE * OZONE	***	***	ns	ns	***	ns	**	*
AGE * VARIETY	***	***	*	ns	*	*	***	*
AGE * TREATMENT	*	***	*	***	**	ns	***	***
OZONE * VARIETY	ns	***	*	**	***	ns	ns	ns
OZONE * TREATMENT	ns	**	**	***	**	ns	**	***
VARIETY * TREATMENT	ns	***	**	***	ns	***	ns	ns
AGE * OZONE * VARIETY	*	ns	ns	*	*	ns	ns	***
AGE * OZONE * TREATMENT	ns	ns	**	***	ns	ns	ns	ns
AGE * VARIETY * TREATMENT	**	*	ns	*	ns	***	*	ns
OZONE * VARIETY * TREATMENT	ns	***	ns	*	ns	ns	ns	**
AGE * OZONE * VARIETY * TREATMENT	ns	*	ns	ns	ns	ns	ns	ns

*, *P* < 0.05; **, *P* < 0.01; ***, *P* < 0.001; ns; not significant.

Antioxidative enzyme tests revealed that plants grown under EO_3_ conditions had much higher levels of enzymatic activity than in AO_3_. In both growth phases, Kashi Sheetal plants had greater APX activity than Kashi Harittima plants (**Figures 5**, **6**). At all ages and in both varieties of Indian bean, there was no significant difference in the activity of the APX enzyme between the N2 and N3 treatments as compared to the control. N1 and N2 treatments showed significantly higher activities of APX in comparison to control in both EO_3_ and AO_3_ conditions. APX activity was significantly higher in the reproductive phase in both the varieties at EO_3_ and AO_3_ conditions. Results of four-way ANOVA revealed that APX varied significantly due to all individual factors and their interactions except age*ozone*variety and age*ozone*treatment (**Table 1**). Similar results were also revealed for activities of other antioxidative enzymes like CAT, GR, and SOD where the activity of these enzymes increased in reproductive stages of growth (**Figures 5**, **6**). N1, N2, and N3 treatments showed significantly higher values of enzymatic activity as compared to the control but there was no significant difference between N2 and N3 treatments. The activity of CAT, GR, and SOD enzymes also increased significantly in the EO_3_ conditions as compared to AO_3_ conditions at all ages and this accounts for both varieties of *Dolichos lablab* L. (**Figures 5**, **6**). GR and SOD showed significant variations for all four individual factors and some of their interactions such as age*variety and age *treatment. CAT showed significant variations for all individual factors and their interactions except age and its interaction with ozone and variety (**Table 1**).

### 3.4 Photosynthetic rate, stomatal gas conductance, and internal CO_2_ concentration

Significantly higher rates of photosynthesis were observed in Kashi Sheetal plants at both ages and in all types of N treatment as compared to Kashi Harittima plants. The plants of both varieties grown in EO_3_ conditions showed a considerable reduction in the rate of photosynthesis in comparison to plants grown in AO_3_ conditions. In the vegetative phase, the Kashi Sheetal showed an 11.2, 25.5, and 26.5% increase at AO_3_ and 8, 24.1, and 27.4% increase at EO_3_ for N1, N2, and N3 treatments, respectively (**Table 2**). There was a 12.3, 26.9, and 28% increase at AO_3_ and a 13, 28, and 30% increase at EO_3_ for N1, N2, and N3 treatments, respectively of the Kashi Harittima variety in the vegetative phase (**Table 2**). Similarly in the reproductive phase, the Kashi Sheetal showed a 14.7, 30.3, and 28.4% increase at AO_3_ and 14.8, 27.8, and 26.5% increase at EO_3_ for N1, N2, and N3 treatments, respectively (**Table 3**). There was an 11.5, 24.2, and 26.3% increase at AO_3_ and a 10.2, 22.7, and 25% increase at EO_3_ for N1, N2, and N3 treatments, respectively of the Kashi Harittima variety in the reproductive phase (**Table 3**). After analyzing the degree of percent changes between the N treatments and control in both varieties, the N2 treatment was the most effective treatment in increasing the rate of photosynthesis. According to the four-way ANOVA results, the rate of photosynthesis varied significantly for all individual factors, and some of their interactions such as age*variety, variety*treatment, and age*variety*treatment (**Table 1**). Similar results were also observed for stomatal gas conductance and internal CO_2_ concentration in both the varieties of Dolichos lablab under AO_3_ and EO_3_ conditions. For both parameters, the Kashi Sheetal variety showed better results in all types of N treatments as compared to Kashi Harittima. The values of stomatal gas conductance and internal CO_2_ concentration were significantly reduced in EO_3_ conditions in comparison to AO_3_ for both varieties at all ages. Stomatal gas conductance and internal CO_2_ concentration varied significantly for all four individual factors and the interactions of age with ozone, variety, and treatment (**Table 1**).

**Table 2:** Effect of N treatment (C, control; N1, recommended N dose; N2, 1.5-times recommended N dose and N3, 2-times recommended N dose) on physiological parameters of *Dolichos lablab* L. under AO_3_ at vegetative stage and reproductive stage.

a) N treatment	Rate of photosynthesis(A; μmolCO_2_ m^-2^ s^-1^)	Stomatal gas conductance(g_s_; mmolCO_2_ m^-2^ s^-1^)	Internal CO_2_(Ci; μmol mol^-1^)
Vegetative Phase			
C	9.8^c^ ± 0.42	132.9^c^ ± 3.71	310.4^c^ ± 4.5
N1	10.7^b^ ± 0.55	139.6^b^ ± 2.48	323.0^b^ ± 2.6
N2	12.3^a^ ± 0.82	155.4^a^ ± 3.03	340.9^a^ ± 3.1
N3	12.4^a^ ± 0.23	154.2^a^ ± 3.11	343.5^a^ ± 2.9
Reproductive Phase			
C	10.2^c^ ± 0.35	136.9^c^ ± 3.69	318.1^c^ ± 3.2
N1	11.7^b^ ± 0.42	147.3^b^ ± 4.5	329.4^b^ ± 2.9
N2	13.3^a^ ± 0.7	160.7^a^ ± 2.99	349.6^a^ ± 3.5
N3	13.1^a^ ± 0.93	159.3^a^ ± 4.02	350.5^a^ ± 4.0
b) N treatment	Rate of photosynthesis(A; μmolCO_2_ m^-2^ s^-1^)	Stomatal gas conductance(g_s_; mmolCO_2_ m^-2^ s^-1^)	Internal CO_2_(Ci; μmol mol^-1^)
Vegetative Phase			
C	8.9^c^ ± 0.31	126.3^c^ ± 2.98	303.4^c^ ± 3.6
N1	10.0^b^ ± 0.64	133.5^b^ ± 3.42	315.7^b^ ± 2.7
N2	11.3^a^ ± 0.7	145.7^a^ ± 3.9	331.6^a^ ± 3.0
N3	11.4^a^ ± 0.85	146.8^a^ ± 2.66	331.0^a^ ± 2.2
Reproductive Phase			
C	9.5^c^ ± 0.7	133.4^c^ ± 2.6	310.4^c^ ± 2.5
N1	10.6^b^ ± 0.58	140.7^b^ ± 4.45	310.4^c^ ± 2.5
N2	11.8^a^ ± 0.67	148.3^a^ ± 2.5	335.9^a^ ± 3.5
N3	12.0^a^ ± 0.55	149.1^a^ ± 3.36	336.1^a^ ± 2.8

Values are mean ± SE. a) Kashi Sheetal, b) Kashi Harittima.

**Table 3:** Effect of N treatment (C, control; N1, recommended N dose; N2, 1.5-times recommended N dose and N3, 2-times recommended N dose) on physiological parameters of *Dolichos lablab* L. under EO_3_ at vegetative stage and reproductive stage. Values are mean ± SE. a) Kashi Sheetal, b) Kashi Harittima.

a) N treatment	Rate of photosynthesis(A; μmolCO_2_ m^-2^ s^-1^)	Stomatal gas conductance(g_s_; mmolCO_2_ m^-2^ s^-1^)	Internal CO_2_(Ci; μmol mol^-1^)
Vegetative Phase			
C	9.1^c^ ± 0.28	124.5^b^ ± 2.66	299.0^c^ ± 4.8
N1	9.9^b^ ± 0.45	129.7^b^ ± 3.78	308.2^b^ ± 6.9
N2	11.3^a^ ± 0.62	144.1^a^ ± 4.4	320.7^a^ ± 5.1
N3	11.6^a^ ± 0.3	145.7^a^ ± 3.15	322.4^a^ ± 4.7
Reproductive Phase			
C	9.4^c^ ± 0.52	127.4^c^ ± 4.2	306.2^c^ ± 4.0
N1	10.8^b^ ± 0.67	130.0^b^ ± 3.91	314.7^b^ ± 5.3
N2	12.0^a^ ± 0.45	148.6^a^ ± 2.58	325.0^a^ ± 3.8
N3	11.9^a^ ± 0.3	149.1^a^ ± 4.56	327.1^a^ ± 6.1
b) N treatment	Rate of photosynthesis(A; μmolCO_2_ m^-2^ s^-1^)	Stomatal gas conductance(g_s_; mmolCO_2_ m^-2^ s^-1^)	Internal CO_2_(Ci; μmol mol^-1^)
Vegetative Phase			
C	8.2^c^ ± 0.42	117.5^c^ ± 3.41	292.5^c^ ± 3.6
N1	9.3^b^ ± 0.57	123.3^b^ ± 2.9	301.6^b^ ± 4.8
N2	10.5^a^ ± 0.39	135.0^a^ ± 4.57	314.2^a^ ± 5.2
N3	10.7^a^ ± 0.66	136.2^a^ ± 3.95	316.1^a^ ± 6.8
Reproductive Phase			
C	8.8^c^ ± 0.44	124.7^c^ ± 4.2	303.5^c^ ± 5.5
N1	9.7^b^ ± 0.8	133.1^b^ ± 3.52	310.6^b^ ± 6.4
N2	10.8^a^ ± 0.35	148.4^a^ ± 4.67	321.4^a^ ± 2.9
N3	11.0^a^ ± 0.61	150.0^a^ ± 3.69	321.9^a^ ± 4.7

### 3.5 Yield

There was a significant reduction in the weight of seeds plant^-1^ for both varieties under EO_3_ conditions as compared to AO_3_ conditions. For the Kashi Sheetal variety, there was a 20.7,34.5, and 35.4% increase at AO_3_ and 26.8, 35.8, and 36.8% increase at EO_3_ for N1, N2, and N3treatments, respectively in comparison to control (**Table 4**). There was a 22.4, 42.6, and 43.8% increase at AO_3_ and a 28.7, 40.4, and 41.2% increase at EO_3_ for N1, N2, and N3 treatments, respectively of the Kashi Harittima variety as compared to control (**Table 4**). There was a significant reduction in the test weight of 1000 seeds for both varieties under EO_3_ conditions as compared to AO_3_ conditions except for the control of both varieties. For the Kashi Sheetal variety, there was a 9.7, 16.1, and 17.3% increase at AO_3_ and 8.01, 12.4, and 12.9% increase at EO_3_ for N1, N2, and N3 treatments, respectively in comparison to control. There was a 14.4, 20.2, and 20.6% increase at AO_3_ and an 18.8, 28.4, and 28.9% increase at EO_3_ for N1, N2, and N3 treatments, respectively of the Kashi Harittima variety as compared to control (**Table 4**). The percent change of all treatments was higher for the Kashi Harittima variety in comparison to the Sheetal variety. Only the N2 and N3 treatments of the Harittima variety were significant for the number of seeds plant^-1^ as compared to the control. All the other values of Sheetal and Harittima varieties for the number of seeds plant^-1^ were found to be insignificant.

**Table 4:** Variations in the number of seeds plant^-1^, wt. of seeds plant^-1^, and test wt. per 1000 seeds of both varieties of *Dolichos lablab* L. grown under AO_3_ and EO_3_.

	Number of seeds plant^-1^		Weight of seeds plant^-1^ (g)		Test wt. per 1000 seeds (g)	
	AO_3_	EO_3_	AO_3_	EO_3_	AO_3_	EO_3_
Kashi Sheetal						
C	70.8 ± 5.3	64.1^ns^ ± 3.2	134.5 ± 11.2	121.7* ± 13.8	405.9 ± 15.9	378.2^ns^ ± 10.4
N1	81.2 ± 4.5	77.2^ns^ ± 2.8	162.4 ± 13.5	154.4* ± 11.6	445.3 ± 14.6	408.5* ± 11.2
N2	96.0 ± 3.9	89.6^ns^ ± 4.3	181.0 ± 12.9	165.3* ± 10.7	471.6 ± 20. 8	425.1** ± 15.0
N3	98.8 ± 6.2	90.0^ns^ ± 5.7	182.2 ± 13.4	166.5** ± 9.2	476.3 ± 19.5	427.0*** ± 16.7
Kashi Harittima						
C	67.2 ± 5.1	56.8^ns^ ± 3 .3	120.9 ± 10.6	112.3* ± 14.8	375.2 ± 14.5	311.8^ns^ ± 12.6
N1	78.4 ± 3.5	70.0^ns^ ± 4.4	148.1 ± 12.1	144.6* ± 17.1	429.3 ± 13.6	370.6* ± 11.9
N2	92.4 ± 4.1	79.6*** ± 6.7	172.5 ± 13.3	157.7** ± 16.3	451.0 ± 17.7	400.5** ± 14.4
N3	93.2 ± 4.0	79.2*** ± 4.1	173.9 ± 12.7	158.6*** ± 10.4	452.5 ± 15.9	402.1** ± 15.3

Values are mean ± SE (ns; not significant; *, P ≤ 0.05; **, P ≤ 0.01; ***, P ≤ 0.001).

## 4 Discussion

Sicard et al. (2017) predicted a rise in O_3_ concentration under all climate change scenarios and identified South Asia as one of the primary O_3_ hotspot regions. Intense irradiance, elevated temperature, and low moisture are the ideal circumstances for O_3_ generation in the Indo-Gangetic plains, where high episodes of O_3_ are common occurrences (Sarkar and Agrawal, 2010). In monitoring studies conducted at the current experimental site between 2002 - 2012, the concentration of O_3_ was clearly on the rise (Tiwari and Agrawal, 2018). The results of the present experiment evidently revealed the presence of high ozone episodes during the reproductive phase of both varieties of *Dolichos lablab* L. The increased O_3_ levels during the reproductive phase can be reasoned by the high temperature in the months of January, February, and March as compared to November and December months. High O_3_ concentrations have also been recorded in earlier experiments conducted at the current experimental location during the same time period (Singh et al., 2018; Yadav et al., 2019).

Out of the two varieties of *Dolichos lablab* L. exposed to ambient and elevated O_3_ concentrations, Kashi Sheetal had higher endogenously generated H_2_O_2_ in its control leaves as compared to Kashi Harittima, as evident through the histochemical assay of H_2_O_2_ localization (**Figure 4**). Related findings have reported that cultivars with higher levels of endogenous H_2_O_2_ are more tolerant than cultivars with lower levels of H_2_O_2_ (Caregnato et al., 2013; Yadav et al., 2019; Gupta and Tiwari, 2020). Caregnato et al. (2013) reported significantly higher H_2_O_2_ localization in the leaves of two varieties of *Phaseolus vulgaris* L. grown in EO_3_ conditions in comparison to AO_3_ conditions. The high H_2_O_2_ localization in the leaves is because of the excessive ROS generated due to severe oxidative stress which happens due to elevated O_3_ fumigation. With an increased dose of nitrogen treatments, there was a significant degree of reduction in the amount of H_2_O_2_ localization in the leaves of both varieties (**Figure 4**). It was reported that H_2_O_2 was_ produced in the leaves of *Cymopsis tetragonoloba* L. Taub. (Cluster bean) was significantly decreased with increasing nitrogen amendment doses (Gupta and Tiwari, 2020). It is suggested that the surplus nitrogen available to the plant gets allocated to improve the antioxidative potential of the plants which results in improved scavenging of the H_2_O_2_ (Podda et al., 2019). It is noted that N2 treatment was found to be sufficient in the management of O_3_ stress under both O_3_ exposure conditions.

**Figure 7:**
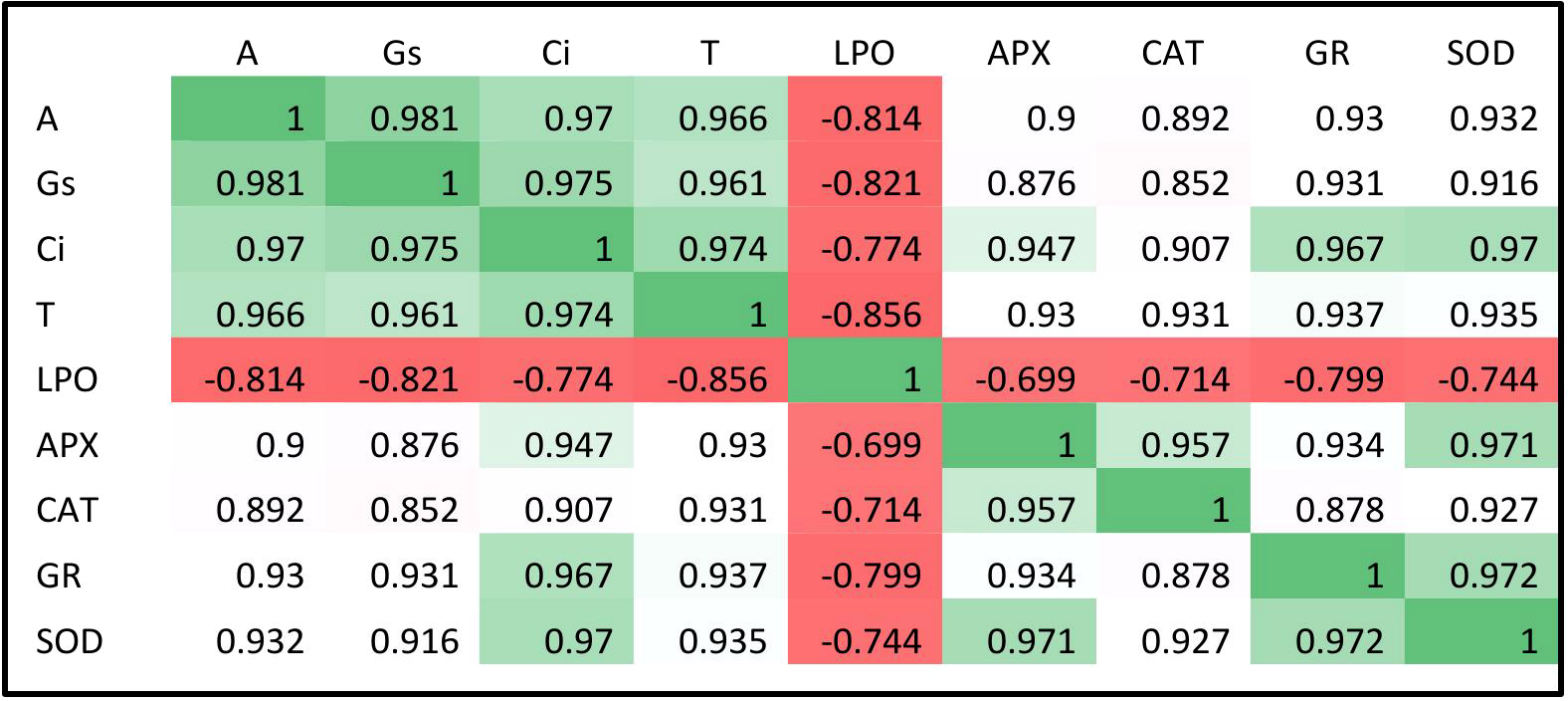
Pearson’s correlation coefficient for physiological and biochemical parameters of two varieties of Hyacinth bean at EO_3_.

Malondialdehyde is an intermediate product that is exclusively produced during the process of membrane lipid peroxidation, so measuring the MDA content gives an approximation of the degree of lipid peroxidation. Biochemical analysis of lipid peroxidation showed higher MDA content in the EO_3_ conditions in both varieties of *Dolichos lablab* L. as compared to AO_3_ at both developmental stages (**Figures 5**, **6**). The findings suggest that greater O_3_ influx under EO_3_ conditions resulted in significant membrane damage in both kinds of Indian beans at both development phases. A 90.2% increase in MDA contents in *Vigna mungo* L. varieties was reported on exposure to EO_3_ conditions as compared to AO_3_ conditions (Dhevagi et al., 2021). Since lipid peroxidation is an important marker of O_3_ stress tolerance (Iglesias et al., 2006), Kashi Sheetal which showed a lesser degree of membrane lipid peroxidation under EO_3_ conditions as compared to Kashi Harittima, was considered to be more tolerant of the two experimental varieties. The level of MDA content in the leaves of both varieties of *Dolichos lablab* L. was significantly reduced upon nitrogen treatments at both the O_3_ concentrations. Similar findings were also reported by Pandey et al. (2018) in which MDA content in the leaves of *Triticum aestivum* L. was significantly decreased with increased nitrogen amendments. Podda et al. (2019) also found that nitrogen treatment had a beneficial impact on O_3_-exposed plants, seeing a substantial decrease in MDA and improved membrane stability after nitrogen treatment at both AO_3_ and EO_3_ conditions. It was observed that Kashi Sheetal produced a lesser amount of MDA in comparison to the Kashi Harittima, at all the nitrogen treatments during both developmental stages, indicating Kashi Harittima to be was less efficient in scavenging the O_3_-induced ROS leading to higher membrane disintegration than the Kashi Sheetal upon nitrogen treatments under both O_3_ exposure conditions. This observation points towards the higher sensitivity of the Kashi Harittima towards ozone stress. Furthermore, there was a negligible difference in the response between the N2 and N3 treatments for both varieties upon both O_3_ exposure conditions, which specifies the sufficiency of N2 treatment in alleviating O_3_ stress in the Indian bean. Pearson’s correlation coefficient test has shown a significant negative correlation of MDA contents with the enzymatic activities, suggesting a reduction in membrane lipid peroxidation of O_3_-stressed plants upon N amendments (**Figure 7**).

**Figure 8:**
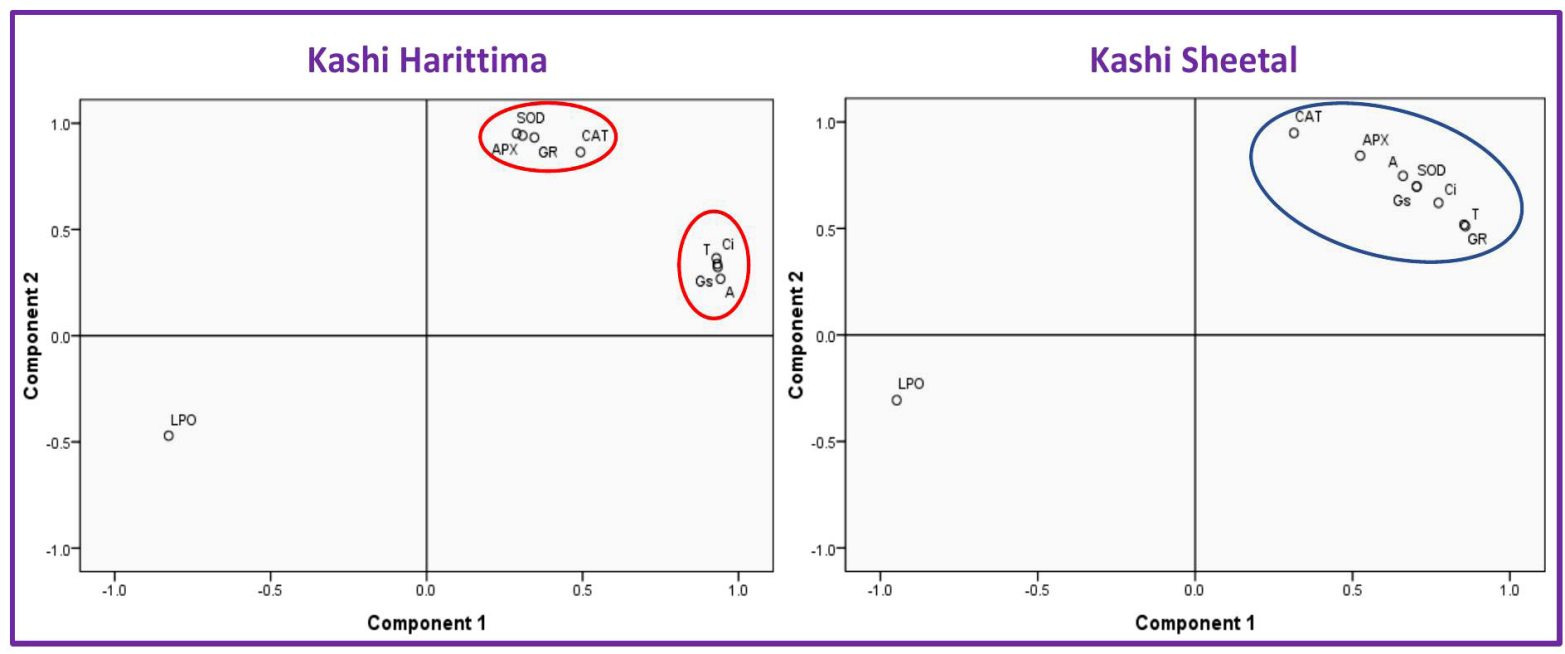
Principle Component Analysis (PCA) showed the association of considered parameters on two different components; i.e, component 1 and component 2. The parameters studied are APX, GR, SOD, CAT, LPO, Ci, g_s, and_ **(A)**_(_ *a*_)_ Association of studied parameters in Kashi Harittima, **(B)** association of studied parameters in Kashi Sheetal.

The presence of an efficient antioxidant defense mechanism allows the higher plants to perceive and decipher ROS signals into necessary cellular responses (Yadav et al., 2019). In the current experiment, it was observed that the endogenous level of antioxidants was higher in Kashi Sheetal as compared to Kashi Harittima, at ambient O_3_ exposure, suggesting high tolerance of O_3_ stress in Kashi Sheetal. All the antioxidative enzymes showed higher activity under EO_3_ conditions as compared to AO_3_ conditions and this applies to both varieties at all developmental stages. Similar findings have also been reported by Pandey et al. (2018) in which the activities of antioxidative enzymes increased under EO_3_ conditions in two cultivars of *Triticum aestivum* L. It has been proved that the sustenance and regeneration of the antioxidative enzymatic pool indicate a strengthened defense response capable of O_3_ detoxification (Caregnato et al., 2013). In the present study, N amendments enhanced the antioxidant activities in both varieties, thereby proving its potential in the management of O_3_ injury. It was observed that the antioxidative system of O_3_ sensitive variety Kashi Harittima responded more positively at elevated O_3_, compared to ambient O_3_. At elevated O_3_, Kashi Harittima was capable of sustaining higher increments in the enzyme activities at both developmental stages, which evidently proves that N amendments were more favorable in encountering O_3_ stress in this variety, as compared to Kashi Sheetal. As per the PCA analysis, it was observed that Kashi Harittima, as the sensitive variety, demonstrated a stronger association of enzymatic anti-oxidants in component 1 as compared with the Kashi Sheetal (**Figure 8**). In the ascorbate-glutathione pathway, APX is the essential enzyme known for scavenging H_2_O_2_ by using ascorbic acid as an electron donor, whereas GR catalyzes glutathione reduction with ascorbate regeneration in plants (Ashraf, 2009). In the present experiment, N amendments intensified the role of APX and GR in inducing the plant’s defense system in both varieties. The higher APX and GR activity can be owed to the greater H_2_O_2_ level in EO_3_ conditions than in AO_3_, thereby ensuring the scavenging of the H_2_O_2_ in order to curtail O_3_ stress. Under stressful circumstances, Almeselmani et al. (2006) also observed an increment in APX and GR activity in the tolerant wheat cultivars HD77 and HD2817. The lower levels of H_2_O_2_ in Kashi Harittima, upon N amendments at elevated O_3_ treatment, can be explained by higher increments in CAT activity. Upon N amendments, whereas, Kashi Harittima was able to uphold the activities of SOD and CAT, in elevated O_3_-treated plants, Kashi Sheetal showed lesser increments in the response of these two enzymes, during the reproductive stage. It is to be noted that with a gradual increase in the dose of nitrogen treatments, there was a significant surge in the activities of the antioxidative enzymes in both varieties of *Dolichos lablab* L. at all growth stages. But the degree of increment of the activities of enzymes was insignificant between N2 and N3 treatments, suggesting the adequacy of N2 treatment in the management of O_3_ stress.

O_3_ exposure is known to adversely affect the physiological parameters of plants, rate of photosynthesis, and stomatal conductance being directly affected (Singh et al., 2014; Tetteh et al., 2016; Dhevagi et al., 2021). This reduced photosynthetic rate caused by elevated ozone may be related to the degradation of the chloroplast structure, which prevents the production of chlorophyll under these conditions (Biswas and Jiang, 2011). In the present study, N supplementation resulted in the improvement of these two parameters in both the varieties of *Dolichos lablab*, at both O_3_ exposure conditions, further justifying the potential role of N amendments in depreciating O_3_ stress. This is further proved by significant correlation coefficient values of different physiological parameters of O_3_-exposed plants upon N amendments (**Tables 2**, **3**). Increased g_s_ upon N amendments have been reported in wheat (Wall et al., 2000), larch (Mao et al., 2014), cluster bean (Gupta and Tiwari, 2020), etc. A decrease in g_s_ along with an increased A upon N amendments and O_3_ exposure in poplar has been reported by Zhang et al. (2018). A comparison of the physiological response of both varieties revealed that upon N supplementation, Kashi Harittima showed higher increments in A and *g_s_*, in comparison to Kashi Sheetal at all ages under both AO_3_ and EO_3_ conditions. Since the O_3_-induced depreciation of cellular performance largely depends upon O_3_ flux, which is directly controlled by stomatal movement (Yadav et al., 2019), increased stomatal conductance in O_3_-exposed plants of Indian bean in the present study, does not account for the reported improvement in the plant’s performance upon N amendments. It has been reported that the application of a higher N dose leads to an increased influx of O_3_, which may lead to the depletion of the antioxidant pool (Harmens et al., 2017; Marzuoli et al., 2018). Non-significant variations in *g_s_* due to O_3_ x variety and O_3_ x treatment interactions (**Table 1**) further prove that *g_s_* does not play any substantial role in the ameliorative effect of N amendments in the experimental plants. These observations lead to the conclusion that the upturn in the rate of photosynthesis upon N amendments in O_3_-exposed plants can be attributed to the enhanced activities of the enzymatic antioxidant pool, thus proving our hypothesis. Increased enzymatic antioxidants upon N implementation, ensure the scavenging of O_3_-induced oxidative entities, thus rendering protection to photosynthetic machinery in the chloroplasts.

The analysis of yield parameters showed a significant reduction in the EO_3_ conditions in comparison to AO_3_ conditions which accounts for both varieties of *Dolichos lablab* L. at all ages of growth (**Table 4**). An earlier study found that the yield of selected varieties of wheat (HUW 234 and 468, HD 3086 and 3118) and black gram (CO6 and VBN 1-8) were markedly reduced under EO_3_ as compared to AO_3_ (Yadav et al., 2019; Dhevagi et al., 2021). Both varieties of the Indian bean exhibited a significant increase in the weight of grains plant^-1^ upon all doses of N amendments (N1, N2, and N3). However, the difference in the magnitude of the increase in yield of N2 and N3 was inconsequential for both varieties indicating the sufficiency of N2 treatment in ameliorating oxidative conditions. Interestingly, the O_3_-sensitive Kashi Harittima variety showed a higher percent increment in the number/weight of seeds plant^-1^ upon N amendments as compared to Kashi Sheetal (**Table 4**). Higher enhancement in the antioxidative enzyme activity and greater increments in the rate of photosynthesis explain the higher yield improvements in Kashi Harittima. A previous experiment also reported similar findings in which an O_3_-sensitive wheat cultivar showed a higher percent increment in response to N amendments as compared to an O_3_-tolerant wheat cultivar under both AO_3_ and EO_3_ conditions (Pandey et al., 2018). The above results prove our first hypothesis that nitrogen treatments assist in strengthening the antioxidative enzyme pool for defense against oxidative stress conditions. In the present study, the analysis of H_2_O_2_ localization, antioxidative enzymes, physiological traits, and yield revealed that the Kashi Sheetal variety outperformed the Kashi Harittima variety in every aspect proving the second hypothesis that the differential response of the varieties to varied O_3_ conditions helps in determining their sensitivity to O_3_ stress. The more positive response of the O_3_-sensitive varieties upon N exposure provides a promising feature and can be used for promoting the farming of O_3_-sensitive varieties, thereby boosting agricultural production.

## 5 Conclusion

This experiment justifies the use of N amendments as an effective measure for the management of O_3_ injury. The higher endogenous levels of H_2_O_2_ accumulation and enzymatic antioxidant contents upon O_3_ exposure have established Kashi Sheetal to be O_3_ tolerant. The interaction between N amendments and O_3_ exposure had a more positive effect on O_3_-sensitive Kashi Harittima, as compared to Kashi Sheetal. Since the increased stomatal conductance upon N fertilization does not restrict the entry of O_3_ in plants, the higher photosynthetic rate, and subsequently yield were maintained by a stimulated enzymatic antioxidative response in plants at both O_3_ exposure conditions. The enzymatic response showed significant variations due to ozone and treatment and ozone and variety interactions, whereas the variations of stomatal conductance were insignificant, proving our theory. A more positive response of Kashi Harittima to N supplementation at both O_3_ exposure conditions can be associated with the sustenance of its higher enzymatic response at the reproductive stage as well, which was more prominent at the EO_3_ condition. The current study proved that N2 treatment (1.5 times recommended dose) was sufficient to partially ameliorate O_3_ stress and that larger nitrogen doses may not be more successful in doing so because they did not confer any further perks on the plant’s growth and development. However, more experiments are required to establish a dose-response relationship between N fertilization and O_3_ exposure doses.

## Data availability statement

The original contributions presented in the study are included in the article/supplementary material. Further inquiries can be directed to the corresponding author.

## Author contributions

Conceptualization, writing-original draft, AS. Data curation, PM. Software, AM. Conceptualization, supervision, ST. All authors contributed to the article and approved the submitted version.
